# Mid-infrared dispersive wave generation in gas-filled photonic crystal fibre by transient ionization-driven changes in dispersion

**DOI:** 10.1038/s41467-017-00943-4

**Published:** 2017-10-09

**Authors:** F. Köttig, D. Novoa, F. Tani, M. C. Günendi, M. Cassataro, J. C. Travers, P. St.J. Russell

**Affiliations:** 1 Max Planck Institute for the Science of Light, Staudtstrasse 2, 91058 Erlangen, Germany; 2 School of Engineering and Physical Sciences, Heriot-Watt University, Edinburgh, EH14 4AS UK

## Abstract

Gas-filled hollow-core photonic crystal fibre is being used to generate ever wider supercontinuum spectra, in particular via dispersive wave emission in the deep and vacuum ultraviolet, with a multitude of applications. Dispersive waves are the result of nonlinear transfer of energy from a self-compressed soliton, a process that relies crucially on phase-matching. It was recently predicted that, in the strong-field regime, the additional transient anomalous dispersion introduced by gas ionization would allow phase-matched dispersive wave generation in the mid-infrared—something that is forbidden in the absence of free electrons. Here we report the experimental observation of such mid-infrared dispersive waves, embedded in a 4.7-octave-wide supercontinuum that uniquely reaches simultaneously to the vacuum ultraviolet, with up to 1.7 W of total average power.

## Abstract (web summary)

Dispersive wave emission in gas-filled hollow-core photonic crystal fibres has been possible in the visible and ultraviolet via the optical Kerr effect. Here, Köttig et al. demonstrate dispersive waves generated by an additional transient anomalous dispersion from gas ionization in the mid-infrared.

## Introduction

Coherent nonlinear interactions between intense laser light and photo-induced plasmas are important in fields such as optical filamentation^1^, laser wakefield acceleration^2^, attoscience (through high harmonic generation^3^) and wideband terahertz pulse generation^4^. While these phenomena have been widely studied in free space and capillary fibres, hollow-core photonic crystal fibres (PCFs) give much better control, for example, over the interaction between a single-cycle optical soliton and a self-induced plasma. Broad-band-guiding hollow-core PCFs come in two main varieties—kagomé-type^5^ and single-ring^6–8^—and offer weak anomalous waveguide dispersion that can be counter balanced by the normal dispersion of the filling gas, enabling well-controlled bright soliton dynamics^9, 10^. Broad-band guidance comes about through anti-resonant reflection (ARR), and as a result ARR-PCFs offer long well-controlled collinear path-lengths that make it possible to obtain single-cycle pulses with microjoule energies via soliton self-compression^11, 12^. Along with the compression process in the fibre, ultrashort dispersive wave (DW) bands can be efficiently emitted on the opposite side of the zero dispersion point (ZDP), in the normal dispersion regime^13–15^.

Recently there has been increased interest in coherent ultrafast sources in the mid-infrared (MIR), driven by applications in time-resolved molecular spectroscopy^16^ and the development of small-footprint ultrafast X-ray lasers^17^. Many efficient MIR sources are based on solid-state materials, employing techniques such as difference frequency generation^18^, optical parametric amplification^19^ and DW emission from solitons in crystals with cascaded quadratic nonlinearities^20^.

The high damage thresholds of ARR-PCFs permits operation even in the strong-field regime^21^. Upon self-compression of the input pulse, the peak intensity reaches a level high enough to partially ionize the gas. The presence of free electrons enables resonant transfer of energy to MIR DWs in the anomalous dispersion region, i.e., on the same side of the ZDP as the pump^22^. This counterintuitive effect can be viewed as being caused by a plasma-induced transient change in the frequency dependence of the refractive index (normally assumed to be independent of optical intensity) that triggers phase-matched emission of MIR DWs.

Here we report the experimental observation of such plasma-induced MIR DW emission. At the same time, the intense nonlinear dynamics in the ARR-PCF create a supercontinuum that is at least (limited by our detection system) 4.7 octaves (1.6 PHz) wide, reaching from 180 nm to 4.7 μm, with up to 1.7 W of total average power. Despite its currently limited efficiency in the MIR, the system has the advantage of simultaneously providing tunable ultrashort pulses in the deep (DUV) and vacuum (VUV) ultraviolet—wavelength ranges where the majority of bio-polymers have electronic resonances (highly relevant for photoemission^23^ and bio-spectroscopy^24^).

## Results

### Experimental results

**Fig. 1:**
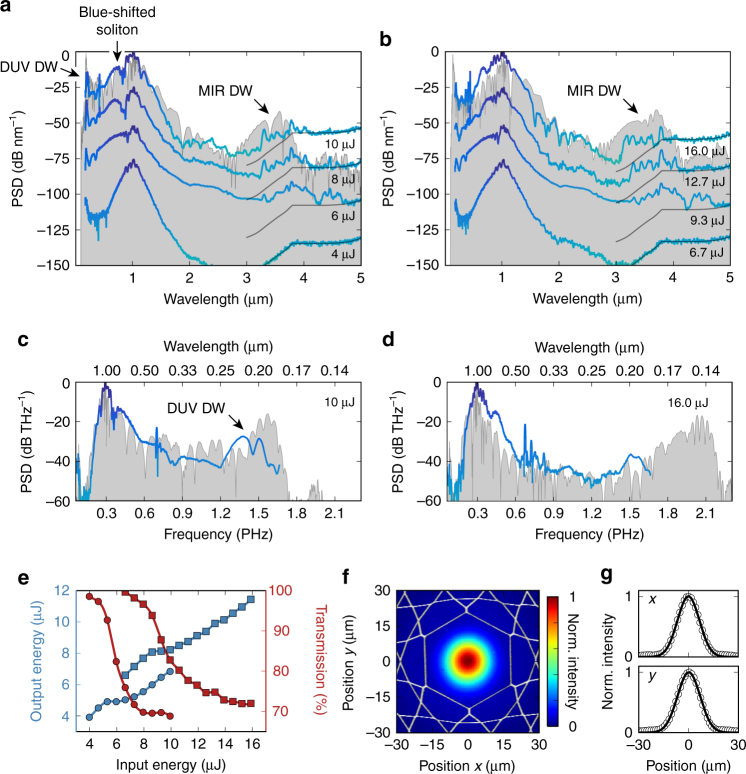
Experimental results. **a** Generated output spectra for wavelengths from 180 nm to 5 μm, when 7 cm of fibre was filled with 4 bar of argon and pumped with pulses of 4, 6, 8 and 10 μJ (the spectra are offset by 25 dB each for clarity). The power spectral density (PSD) is normalized to the peak of the spectra. The PSD from −100 dB to 0 is mapped to a colour gradient from green to blue. At wavelengths longer than 900 nm, the spectra are corrected for the theoretical relative responsivity of the detectors (“Methods” section), but are otherwise intensity-uncalibrated. This leads to a wavelength-dependent noise level (indicated with black lines between 3 and 5 μm) that follows the correction curve for the detector response. Below 200 nm, the spectrum is intensity-uncalibrated. In the vicinity of 4.25 μm, absorption by carbon dioxide in the 2-m-long detection path in air reduces the signal. The relative humidity was ~30%, so that water absorption was negligible in the spectral region of interest. The numerically simulated spectrum (“Methods” section) at 9.7 µJ is shown by the curve under-shaded in grey. Dispersive waves (DWs) are emitted in the deep ultraviolet (DUV) and mid-infrared (MIR). **b** Same as in **a**, but for 30 bar of neon and 15.7 μJ for the simulation. **c**, **d** Spectra versus frequency corresponding to **a**, **b**, scaled by *λ* ^2^/*c* (*λ* is wavelength and *c* the speed of light in vacuum) to represent the correct frequency-scaled PSD. **e** Output energy (blue) and overall transmission (red) of the fibre (including coupling efficiency into the fibre) for the two cases in **a** (circles) and **b** (squares). **f** Near-field optical micrograph of the output mode for wavelengths longer than 3.1 μm, when the fibre was filled with 4 bar of argon and pumped with 6.6 μJ pulses, with the microstructure of the kagomé-photonic crystal fibre superimposed. The intensity is normalized to the maximum value. **g** Normalized intensity profiles in orthogonal directions through the centre of the mode in **f**. The dots are measured points and the lines Gaussian fits

The underlying soliton–plasma interaction takes place in a noble-gas-filled kagomé-type ARR-PCF (36 μm core diameter, 7 cm long), which is pumped by pulses of duration 27 fs and central wavelength 1030 nm, with energies up to 16 μJ and a repetition rate of 151 kHz (“Methods” section). When the fibre is filled with 4 bar of argon, the ZDP is located at 517 nm and the 1030 nm pump pulses lie in the anomalous dispersion region where they are subject to soliton dynamics. For the range of input energies (4–10 μJ), the soliton order lies between 3.3 and 5.2, well below the modulational instability regime^25^, so that coherent soliton fission dominates the dynamics and high self-compression purity is achieved^26^. For input energies greater than 6 μJ, the higher-order input solitons strongly self-compress in the fibre, generating a broadband supercontinuum spanning from 180 nm to 4.7 μm (Fig. 1a). A DUV DW is emitted at a phase-matched wavelength of ~200 nm, with up to 35 nJ pulse energy (5.3 mW of average power, corresponding to 0.6% of the total output). According to numerical simulations (“Methods” section), photoionization in the vicinity of the point of maximum temporal compression creates plasma densities exceeding 5 × 10^17^ cm^−3^, resulting in a transient change in the dispersion that gives rise to phase-matched generation of a second DW on the long-wavelength side of the continuum tail. The bandwidth of this MIR DW extends from ~3.3 to 4 μm, with up to 1 nJ pulse energy (0.15 mW of average power, corresponding to 0.02% of the total output). Its spectral position is in good agreement with the simulations, verifying the underlying phase-matching mechanism (a plasma density of 5.5 × 10^17^ cm^−3^ yields phase-matching to MIR DWs at 3.5 μm in both 4 bar of argon and 30 bar of neon).

The MIR DW is accompanied by blue-shifting solitons at wavelengths below 800 nm—a typical feature of pulse propagation in the presence of photoionization^21^. The losses associated with the generation of free electrons are apparent in the measured output energy (Fig. 1e), the transmission dropping strongly after the onset of soliton blue-shifting and MIR DW emission. At the same time, bright recombination luminescence is visible through the side of the fibre (Supplementary Note 1).

To investigate how phase-matching to MIR DWs is affected by gas dispersion and nonlinearity, as well as input pulse energy and intensity at the self-compression point, the fibre was filled with the lighter noble gas neon at 30 bar. This shifts the ZDP to 482 nm. For soliton orders close to those in the previous argon experiment (3.2–5, corresponding to input energies between 6.7 and 16 μJ), very similar dynamics were observed (Fig. 1b). Clear MIR DWs are emitted also in this case, with pulse energies up to 1.3 nJ (0.2 mW of average power, corresponding to 0.016% of the total output). Despite the different gas dispersion and nonlinearity, and despite higher input energies and hence higher intensities, the bandwidth of the MIR DWs was similar to that seen in the argon-filled fibre. This is due to the dominance of the plasma contribution in phase-matching, which is analysed in more detail in the next sections.

The signal at wavelengths longer than the MIR DW is caused by the tail of the continuum, and is seen only when the point of maximum temporal compression lies close to the output fibre end. Since it disappears at higher input energies, when compression occurs earlier in the fibre, it is unlikely to be related to the MIR DW. This is supported by numerical simulations (Fig. 3). When the MIR DWs first appear (at input energies of 6 and 9.3 µJ in the experiments with argon and neon, respectively), they are approximately at the −40 dB level with respect to the pump and are at least 13 dB stronger than the surrounding continuum. At the highest input energies, where the continuum tail recoils more strongly, the MIR DWs are more than 20 dB stronger than the surrounding continuum. Since the fibre was designed with thin core walls (~200 nm), so as to provide optimal guidance from the UV to the pump wavelength (1030 nm), its loss increases rapidly at longer wavelengths^27^. In the experiments, this leads to a ~10 dB decrease in the MIR DW signal when the input power is increased to its maximum value and the DW emission occurs earlier in the fibre. However, these propagation losses in the MIR can easily be minimized by bringing the MIR DW emission point close to the output fibre end, either by optimizing the fibre length or by adjusting the input energy. In both the experiments and the simulations, the MIR DWs are spectrally structured. In the simulations, which assume a perfect fibre with smooth capillary-like dispersion and wavelength-independent low loss, this is due to the emission dynamics. In the experiments, the influence of the real fibre structure further contributes to this. In particular, spectral features that are present for different input energies can be seen in both the argon and neon-filled fibre. Data from finite-element modelling (FEM) can be used to approximate the real fibre in pulse propagation simulations. However, since reliable FEM analysis of the kagomé-PCF in the MIR is difficult because of its complex cladding structure, we performed additional experiments in a single-ring ARR-PCF, which has a much simpler cladding structure that facilitates accurate FEM. The corresponding experimental results and simulations, which also qualitatively apply to the kagomé-PCF, are shown in Supplementary Note 2. From the extended analysis, it is evident that the additional spectral features in the MIR are due to local changes in dispersion in the real fibre structure, which occur at longer wavelengths when the light is more and more weakly confined to the core, and also at core-wall resonances at shorter wavelengths (these are responsible for the narrowband emission at ~430 nm or 0.7 PHz). While these resonances structure the spectrum, DW emission in the MIR does not occur when ionization is switched off in the simulations. Since the impact of the real fibre structure is therefore not fundamental for the physical process of plasma-induced phase-matching to MIR DWs, a simplified resonance-free dispersion model is justified. In this case, the phase-matching can be described with a simple analytical model (below) that provides valuable physical insight into the conditions for the DW emission and does not require FEM analysis of the fibre. Thus, while the overall dynamics are well reproduced by the simulations using the idealized fibre model, full quantitative agreement cannot be expected, also because effects such as the accidental excitation of higher-order modes, nonlinear absorption in the gas and the influence of the high repetition rate^28^ (151 kHz) are not included in the simulations.

**Fig. 2:**
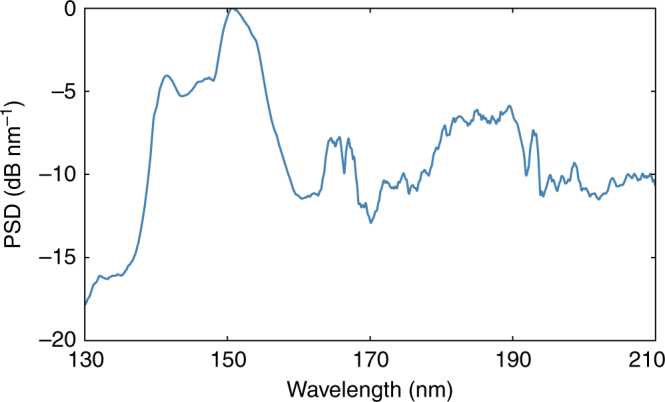
Vacuum ultraviolet dispersive wave emission. Vacuum ultraviolet spectrum (intensity-calibrated) for 15 cm of an identical fibre filled with 30 bar of neon, measured in a separate experiment at ~9 µJ input energy. The power spectral density (PSD) is normalized to the peak of the spectrum

Due to the weaker dispersion of neon at short wavelengths, the DW phase-matching wavelength shifts down to 140 nm in the VUV when the fibre is filled with 30 bar of neon^15^. Owing to the complexity of measurements in the VUV, which must be performed under high vacuum, it was not possible with the existing set-up to make simultaneous measurements in the VUV. Instead, we made use of a previous set of experiments (same system, same fibre, also filled with 30 bar of neon, but with a length of 15 cm instead of 7 cm (Fig. 2)).

For DW emission in the VUV, a massive temporal shock is required (adiabatic pulse compression is insufficient), which for our experimental parameters occurs after only a few centimetres of propagation. It is therefore highly likely that VUV DWs are emitted also in the current experiment, a conclusion that is strongly supported by the observed dynamics (in particular, the shock and the subsequent spectral recoil that generates the MIR DWs). Including these VUV measurements, the supercontinuum spectrum is 5.1 octaves (2.1 PHz) wide.

Although the kagomé-PCF is not single-mode, excitation of higher-order modes can be minimized by careful launch alignment. The MIR near-field profile of the fibre mode at the output face (Fig. 1f, g) has a near-perfect Gaussian shape, with no noticeable higher-order mode content.

Simulations predict that the broad MIR DW band has a spectrally flat phase^22^. Assuming transform-limited pulses with the same spectrum and energy as measured, a pulse of duration ~29 fs (sub-3-cycle) with a peak power exceeding 20 kW appears feasible (Supplementary Note 3).

### Numerical simulations

**Fig. 3:**
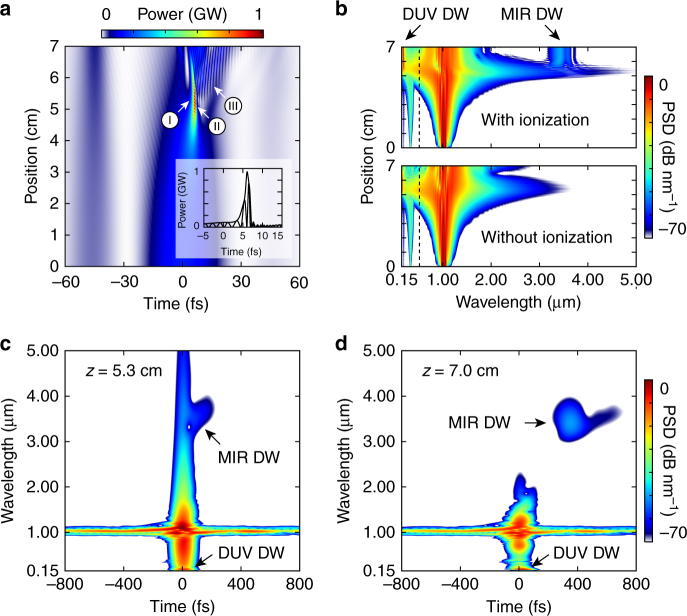
Numerically simulated pulse propagation and time-frequency analysis. Fibre filled with 4 bar of argon, 6 μJ input pulses (measured using frequency-resolved optical gating). **a** Evolution of the temporal pulse shape with propagation distance. The inset shows the pulse at the maximum temporal compression point. (I)—maximum temporal compression point. (II)—shock front. (III)—deep ultraviolet dispersive wave (DUV DW). The pedestal is caused by nonlinear compression of the initially ~300-fs-long pulses from the pump laser (“Methods” section). It is also visible in **c**, **d**. **b** Evolution of the power spectral density (PSD, normalized to the peak of the spectrum) with propagation distance, when ionization is included and when it is switched off (the corresponding evolution of the temporal pulse shape is shown in Supplementary Note 4). The dispersion is anomalous on the long-wavelength side of the zero dispersion point (dashed line). Mid-infrared (MIR) DW emission disappears when ionization is switched off. **c** Spectrogram of the pulse at 5.3 cm (maximum temporal compression point), calculated using a 50-fs-long Gaussian gate pulse. **d** Spectrogram of the pulse at 7 cm (fibre output)

Figure 3 shows the simulated pulse propagation (“Methods” section), when the fibre is filled with 4 bar of argon and pumped with 6 μJ pulses. At the maximum temporal compression point (Fig. 3a, ~5.3 cm), the soliton is self-compressed to a duration of 1.5 fs (full-width at half-maximum), with less than one optical cycle under the intensity envelope. The spectrogram of the pulse at this point (Fig. 3c) confirms soliton self-compression, yielding a near-transform-limited pulse. Upon further propagation, the optical shock effect at the trailing edge of the pulse strongly enhances the short-wavelength side of the spectrum, and energy is efficiently transferred to a phase-matched DW in the normal dispersion region at ~200 nm (Fig. 3b). At the maximum temporal compression point, the peak power reaches the GW level and the intensity rises above 2 × 10^14^ W cm^−2^, resulting in significant plasma generation in the non-perturbative strong-field regime (the Keldysh parameter^29^ *γ* ~ 0.6 at this point). The spectral recoil that accompanies DUV DW emission enhances the long-wavelength side of the spectrum^30, 31^, and the generated plasma densities (of order 5 × 10^17^ cm^−3^) allow phase-matching to a MIR DW in the wavelength range 3–4 μm, in the anomalous dispersion region. Due to its large group velocity mismatch with respect to the pump soliton, the MIR DW has detached from the main pulse by the time it reaches the fibre output (Fig. 3d), underlining its linear-wave character.

### Theory

**Fig. 4:**
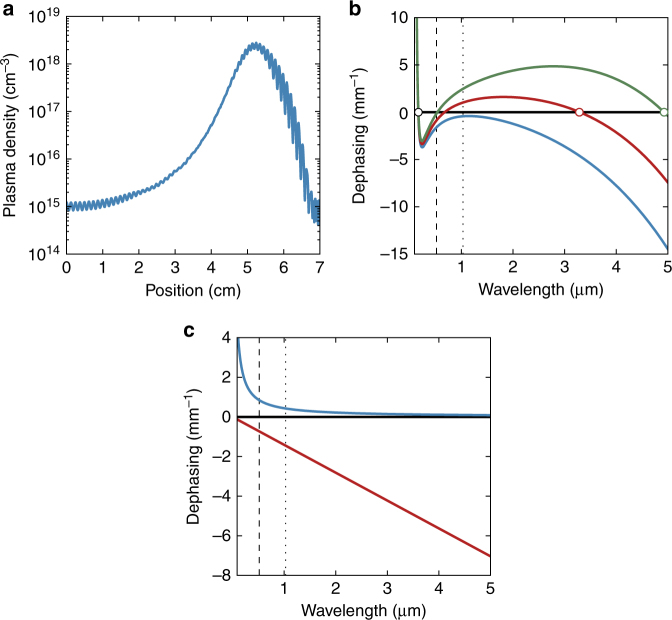
Plasma density evolution and dispersive wave phase-matching. **a** Evolution of the plasma density with propagation distance for the simulation in Fig. 3. As the pulse propagates along the fibre, the carrier-envelope phase (global phase^47^) drifts and the field-dependent ionization rate dithers accordingly, creating ripples in the evolution of the plasma density. **b** Dephasing Δ*β* according to Eq. (1) between a soliton with 1030 nm central wavelength (dotted line) and 1 GW peak power and dispersive waves for the case when the fibre is filled with 4 bar of argon, without plasma (blue) and with plasma densities of 5 × 10^17^ cm^−3^ (red) and 10^18^ cm^−3^ (green). Phase-matching to dispersive waves is satisfied at zero dephasing (circles). The dashed line denotes the zero dispersion point. **c** Contributions of the optical Kerr effect (blue) and plasma polarization (red, plasma density 5 × 10^17^ cm^−3^) to the nonlinear soliton propagation constant

Phase-matching between solitons and linear waves requires momentum conservation, i.e., Δ*β*(*ω*) = *β*(*ω*) − *β*_sol_(*ω*) = 0, where *ω* is frequency and *β* and *β*_sol_ are the propagation constants of linear waves and solitons. An analytical model that includes the contribution of plasma polarization to the nonlinear soliton propagation constant takes the form^22^:

1 $$\Delta \beta \left( \omega \right) = \beta \left( \omega \right) - \left( {{\beta _0} + {\beta _1}\left[ {\omega - {\omega _0}} \right] + \gamma {P_{\rm{P}}}\frac{\omega }{{{\omega _0}}} - \frac{{{\omega _0}}}{{2{n_0}c}}\frac{\rho }{{{\rho _{{\rm{cr}}}}}}\frac{{{\omega _0}}}{\omega }} \right),$$

where *β*_0_ and *β*_1_ are the propagation constant and inverse group velocity at the soliton centre frequency *ω*_0_, *γ* is the pressure-dependent nonlinear fibre parameter^9^ at *ω*_0_, *P*_P_ the soliton peak power, *n*_0_ the linear refractive index at *ω*_0_, *ρ* is the plasma density and *ρ*_cr_ the critical plasma density (at which the plasma becomes opaque at *ω*_0_) and *c* is the speed of light in vacuum. The last two terms on the right-hand side of Eq. (1) originate from the Kerr effect and plasma formation. While the Kerr effect is relevant at high frequencies due to its dispersive correction *ω*/*ω*_0_, the plasma polarization, which goes as *ω*_0_/*ω*, becomes dominant at low frequencies (Fig. 4c). As a result, the phase-matching point for DW emission in the UV can be tuned via the gas dispersion (Figs. 1 and 2) and, to some extent, the soliton peak power, while the plasma density has only a minor influence. In the MIR, however, the gas dispersion and the Kerr effect are comparatively weak, so that phase-matching is governed by both the fibre dispersion and the plasma density created upon self-compression of the input pulse as it propagates along the fibre (Fig. 4a). The bandwidth and position of the MIR DWs are therefore similar in the experiments with argon and neon (Fig. 1).

As illustrated in Fig. 4b, without any plasma contribution, phase-matching to DWs is only possible in the DUV, on the opposite side of the ZDP^30^. For plasma densities greater than 1.2 × 10^17^ cm^−3^, two additional phase-matching points appear, one of which lies in the MIR, progressively shifting to longer wavelength with increasing plasma density. For typical plasma densities reached during self-compression, e.g., 5 × 10^17^ and 10^18^ cm^−3^, phase-matching to MIR DWs is satisfied at 3.3 and 4.9 μm, respectively. While perfect phase-matching is only achieved at zero dephasing, the shallow wavelength-dependence of the dispersion in the gas-filled kagomé-PCF guarantees cm-scale coherence lengths *L*_coh_ = 2π/|Δ*β*| for broadband DW emission in the UV and MIR (e.g., for the parameters given above, with a plasma density of 5 × 10^17^ cm^−3^, the coherence lengths for DW emission exceed 1 cm over bandwidths of 16 THz in the MIR (relative bandwidth of 18%) and 87 THz in the DUV (relative bandwidth of 5%)).

**Fig. 5:**
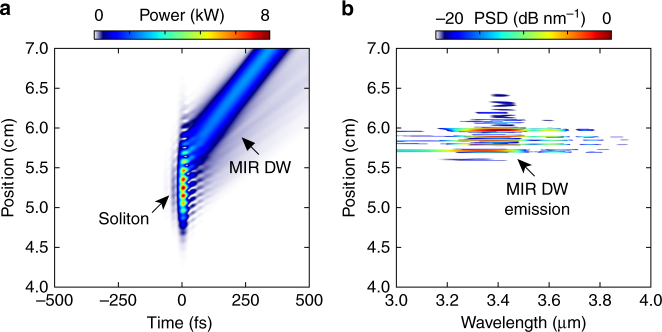
Dynamics of mid-infrared dispersive wave emission. **a** Evolution of the temporal pulse shape with propagation distance, bandpass filtered between 3 and 4 μm, for the simulation in Fig. 3. **b** Power spectral density (PSD, normalized to the peak of the spectrum) of a dispersive wave (DW) generated according to Eq. (2). The central wavelength of the soliton was determined from the first-order centroid of its spectrum. The temporal walk-off between the soliton (central wavelength ~750 nm at the mid-infrared (MIR) DW emission point) and the MIR DW (~3.4 μm) is 33 fs mm^−1^, yielding a gain length of ~1 mm

As phase-matching to MIR DWs is not static, but varies dynamically with the plasma density (Fig. 4), the emission point of the MIR DW does not necessarily coincide with the point of maximum spectral broadening (Fig. 5). Instead, the interplay between spectral overlap with the pump soliton and phase-matching governs the energy transfer to the MIR DW. In the limit of no pump depletion (a good approximation given the low conversion efficiency), the power spectral density of the generated DW takes the form^32^:

2 $${S_{{\rm{DW}}}}\left( {{\omega _{{\rm{DW}}}}} \right) \propto {S_{\rm{P}}}\left( {{\omega _{{\rm{DW}}}}} \right){\rm{sin}}{{\rm{c}}^2}\left( {\frac{{\Delta \beta \left( {{\omega _{{\rm{DW}}}}} \right){L_{{\rm{gain}}}}}}{2}} \right),$$

where *S*_DW_ and *S*_P_ are the power spectral densities of the DW and pump soliton and Δ*β* is the dephasing according to Eq. (1). For the MIR DW, the gain length *L*_gain_ is limited to the mm-scale by temporal walk-off between the pump soliton and the generated DW (Fig. 3c, d). The walk-off length based on group velocity mismatch for pulses with frequencies *ω*_1_ and *ω*_2_ and duration *τ* is given by *L*_walk-off_ = *τ*/|*β*_1_(*ω*_1_) − *β*_1_(*ω*_2_)|, where *β*_1_ = ∂*β*/∂*ω* is the inverse group velocity.

Although the peak power of the soliton, in the spectral range between 3 and 4 μm, is strongest at the point of maximum spectral broadening, the MIR DW is emitted at a point slightly further along the fibre. At the point of maximum spectral broadening, the plasma density exceeds 10^18^ cm^−3^, shifting the phase-matching point of the MIR DW to beyond 5 μm, where the spectral overlap with the pump soliton is low. For the given fibre, MIR DW emission mainly occurs for plasma densities of ~5 × 10^17^ cm^−3^, when the balance between phase-matching and spectral overlap with the pump soliton is such that there is substantial parametric gain.

## Discussion

Soliton–plasma interactions in the single-cycle regime permit emission of DWs in the MIR spectral region. The MIR DWs lie in the anomalous dispersion region, on the same side of the ZDP as the pump solitons, where phase-matching is only possible through the transient plasma correction to the soliton propagation constant. Within a 4.7-octave-wide supercontinuum that reaches into the VUV, MIR DWs are generated in the wavelength range from 3 to 4 μm.

Experiments and simulations show that DW emission is less efficient in the MIR than in the UV. There are two reasons for this. First, the optical shock effect strongly broadens the spectrum towards shorter wavelengths, whereas the spectral power of the soliton is weaker in the MIR as a result of less efficient spectral recoil^30^. Second, the high plasma density at the point of maximum compression impairs phase-matching and prevents efficient transfer of energy to DWs in the MIR; this could be mitigated by balancing spectral broadening and plasma generation to maintain phase-matching over a longer distance. For example, simulations show that the energy in the MIR DW can be enhanced when a comparatively weak second pulse at a different wavelength is co-launched into the fibre. For parameters readily accessible in the experiment (fundamental pulse at 1030 nm with 30 fs and 6 μJ and an easily generated second harmonic (515 nm) pulse with 30 fs and 2 μJ), simulations predict that the energy in the MIR DW increases by almost a factor of six. This suggests that 10-nJ-level pulses in the MIR should be feasible, in which case the peak power could exceed 200 kW.

The results raise the prospect of a novel all-fibre-based source of ultrashort MIR pulses that complements state-of-the-art fibre laser technology^33, 34^ and could be useful, e.g., for pumping soft-glass fibres for multi-octave MIR supercontinuum generation^35–37^. Even though the experimental parameters were not optimized for emission of UV DWs, up to 5.3 mW of average power was generated at ~200 nm, so that the system could be useful for spectroscopy applications or ultrafast pump-probe experiments all the way from the VUV to the MIR^38–40^.

## Methods

### Experimental set-up

The experimental set-up consists of an ultrafast fibre laser, emitting ~300 fs pulses at 1030 nm with 25 μJ energy at 151 kHz repetition rate, and two gas-filled ARR-PCF stages^41^ (Supplementary Methods). In the first stage the laser pulses are spectrally broadened by self-phase modulation in a 19-cm-long single-ring ARR-PCF with 49 μm core diameter filled with 32 bar of argon. Chirped mirrors provide a total of −2100 fs^2^ group delay dispersion to compensate for the positive chirp induced by self-phase modulation and the material dispersion of the optical elements up to the input of the second fibre. Since a fixed negative group delay dispersion is introduced after the first fibre, the duration and phase of the compressed pulses can be fine-tuned by varying the input energy to the first fibre (and hence control the bandwidth generated through self-phase modulation), resulting in 27-fs-long pulses at the input to the second fibre. In the second stage, a 7-cm-long kagomé-PCF with 36 μm core diameter is used, filled either with 4 bar of argon or 30 bar of neon. The loss of this fibre is ~1 dB m^−1^ at the pump wavelength (1030 nm) and below ~5 dB m^−1^ over the wavelength range from 450 nm to 1.75 μm. Launch efficiencies into the fibres in both stages exceeded 90%.

### Measurements

The output of the second fibre was collimated with an aluminium-coated off-axis parabolic mirror and either sent to a thermal power metre to measure the total output power (corrected for the transmission through the magnesium fluoride window of the gas cell and the reflectivity of the parabolic mirror) or to two different spectrometers: a fibre-coupled silicon CCD spectrometer (intensity-calibrated between 200 and 1100 nm) and a monochromator equipped with a lead sulphide detector (Oriel 70341, for 900 nm to 3.8 μm) and a lead selenide detector (Oriel 70344, for 3.8–5 μm). The spectra of the two spectrometers were merged at a wavelength of 900 nm and corrected for the theoretical relative responsivity of the detectors. During a wavelength scan with the monochromator, various order-sorting filters were used to suppress higher diffraction orders of the gratings. During supercontinuum generation, the residual pump signal is still strong. To verify that it does not cause artefacts in the measured spectrum, both fibres were evacuated to eliminate spectral broadening and wavelength scans were performed over the entire spectral range (900 nm–5 μm) using only one order-sorting filter at a time. In these scans, the strong pump signal was blocked below noise level (>60 dB rejection) and no signal was measured over the entire spectrum. During supercontinuum generation, further long-pass filters (cut-on wavelengths 3.1 and 3.6 μm) were inserted in addition to the order-sorting filters of the spectrometer to verify that the spectrum was free from artefacts.

For further measurements, the parabolic mirror was replaced with a plano-convex magnesium fluoride lens. The DUV DW was spatially separated using a calcium fluoride prism and its power measured with a silicon diode power metre and corrected for the transmission of the gas cell window, lens and prism. The MIR DW above 3.1 μm was filtered with two identical long-pass filters and its power measured with a thermal power metre and corrected for the transmission of the gas cell window, lens and the two long-pass filters. For wavelengths longer than 3.1 μm, the optical near-field profile of the fibre mode at the output was imaged using an indium antimonide camera.

For measurements in the VUV, the output of the gas cell was connected to a vacuum spectrometer equipped with an intensity-calibrated silicon photodiode. The spectral response of the system was determined from the transmission of the gas cell window, the grating efficiency of the spectrometer and the response of the photodiode.

### Numerical simulations

Pulse propagation was numerically simulated using a single-mode unidirectional field equation^42^, including photoionization with the Perelomov, Popov, Terent’ev ionization rates^43^, modified with the Ammosov, Delone, Krainov coefficients^44^ (Supplementary Methods). The dispersion of the fibre (36 μm core diameter, ~200 nm core-wall thickness, filled either with 4 bar of argon or 30 bar of neon) was modelled using a simple capillary model^45^, modified by a wavelength-dependent effective core radius that allows an accurate estimate of the modal refractive index at longer wavelength^46^. The *s*-parameter in this modified capillary model was set to *s* = 0.08, a value that was determined by finite-element modelling of an idealized fibre with the same structural parameters. Since the parameters of the input pulse were measured by frequency-resolved optical gating, there were no free parameters in the simulations.

### Data availability

The data that support the findings of this study are available from the corresponding author upon reasonable request.

## Electronic supplementary material

Supplementary Information
