# Supplementary material for: Mid-infrared dispersive wave generation in gas-filled photonic crystal fibre by transient ionization-driven changes in dispersion
Source: Nat Commun. 2017 Oct 9;8:813. doi: 10.1038/s41467-017-00943-4 (PMC5634423; doi:10.1038/s41467-017-00943-4)
Supplement: Supplementary file 1 — Supplementary Information [file 41467_2017_943_MOESM1_ESM.pdf]

## Supplementary Note 1

Due to plasma generation inside the fibre, bright recombination luminescence is scattered through the side of the fibre, showing the characteristic emission lines of singly-ionized argon (Supplementary Fig. 1) or neon. Distinct emission lines appear at the onset of soliton blue-shifting and mid-infrared dispersive wave emission, indicating the strong correlation of these phenomena with the presence of a plasma inside the fibre.

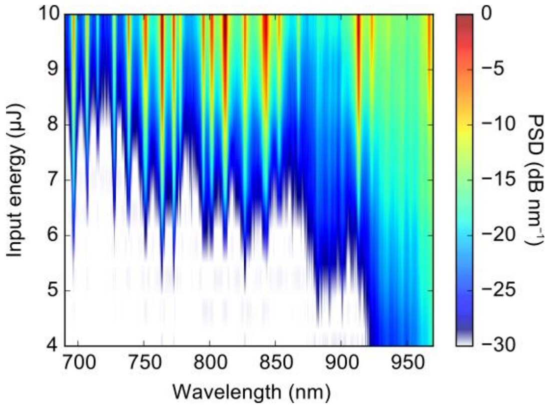


**Supplementary Figure 1 | Side-scatter signal of the fibre.** Power spectral density (PSD, normalized to the peak of the spectrum) of the fibre side-scatter signal, measured via 2*f* imaging onto a fibre-coupled silicon CCD spectrometer, when the fibre was filled with 4 bar of argon. Part of the supercontinuum background comes from light that is reflected at the output window of the gas cell and scattered inside. The side-scatter is measured at ~2 cm from the output fibre end, hence the measured recombination luminescence becomes stronger when the maximum temporal compression point (where the highest plasma densities are created) moves towards the input fibre end with increasing input energy.

## Supplementary Note 2

To investigate mid-infrared dispersive wave (MIR DW) emission in a different fibre, we performed experiments in a single-ring photonic crystal fiber (PCF) with 29 µm core diameter (Supplementary Fig. 9d). We filled a 7-cm-long length of this fibre with 30 bar of neon and pumped it with 6 µJ pulses. In this case, MIR DW emission was observed beyond ~3 µm, with the spectral centroid located at ~3.6 µm (Supplementary Fig. 2a). The MIR DW emission in the kagomé-PCF (36 µm core diameter) is shown for comparison in Supplementary Fig. 2b. In the kagomé-PCF, the spectral centroid is located at ~3.9 µm. The shorter wavelength of the MIR DW emission band in the single-ring PCF compared to the kagomé-PCF is due to its smaller core diameter, which leads to phase-matching at shorter wavelengths.

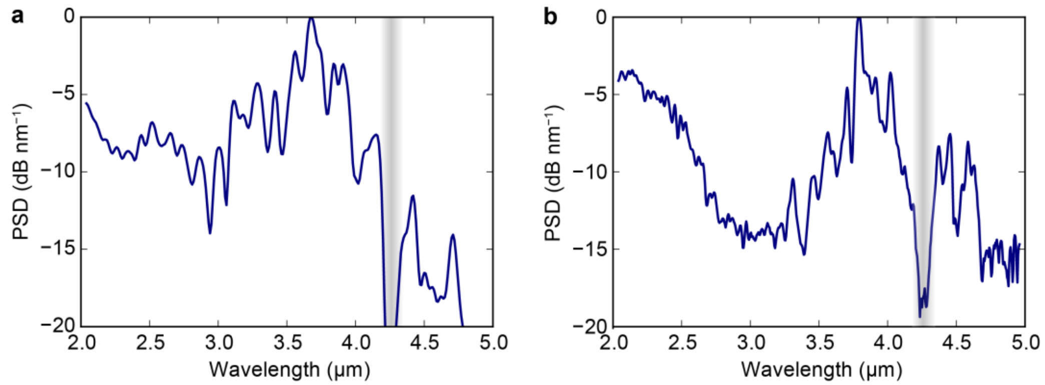


**Supplementary Figure 2 | Mid-infrared spectrum and dispersive wave emission. a,** Single-ring photonic crystal fibre (29 µm core diameter, 6 µJ input energy) and **b,** kagomé-photonic crystal fibre (36 µm core diameter, 10 µJ input energy). Both fibres are 7 cm long and filled with 30 bar of neon. The power spectral density (PSD) is normalized to the peak of the spectra. The different dynamic range with respect to the noise level is due to a different level of attenuation before the spectrometer in the two experiments. The dips in the vicinity of 4.25 µm (grey-shaded) are due to absorption of carbon dioxide in the measurement path in air.

Since reliable finite element modelling (FEM) analysis of the kagomé-PCF in the MIR (where the light is more and more weakly confined to the core) is difficult because of its complex cladding structure, we analyse the impact of the real fibre structure based on the single-ring PCF, which has a much simpler cladding structure that facilitates FEM analysis. Supplementary Fig. 3 shows the modal refractive index and loss of this fibre derived from FEM. Apart from the anti-crossing at the core-wall resonance at ~515 nm, the modal refractive index also exhibits modulations at longer wavelength (>2.8 µm). While the predicted confinement loss is low at the pump wavelength (1030 nm), it increases rapidly at the core-wall resonance and beyond ~1.7 µm.

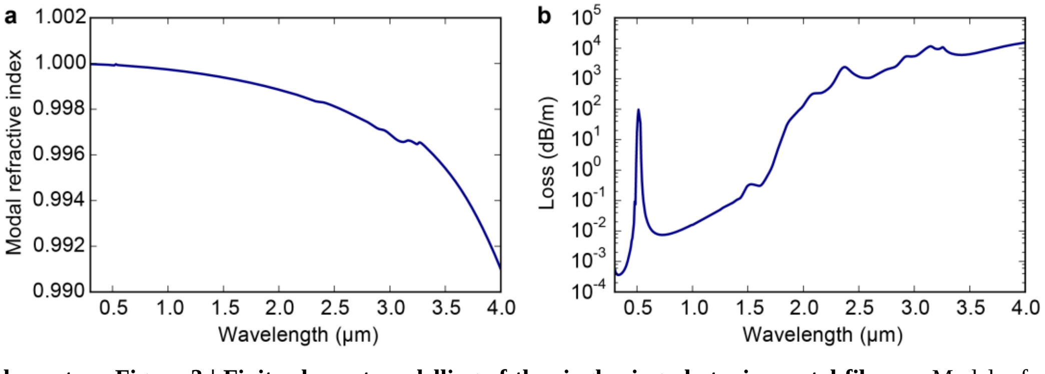


**Supplementary Figure 3 | Finite element modelling of the single-ring photonic crystal fibre. a,** Modal refractive index of the fundamental core mode (evacuated fibre). **b,** Confinement loss of the fundamental core mode.

To investigate the influence of the dispersion of the actual fibre structure (modal refractive index calculated by FEM) compared to the idealized case (modal refractive index according to a modified resonance-free capillary model (Supplementary Eqs. (1) and (2)) fitted to the FEM data), we consider

pumping the fibre with a 27-fs-long Gaussian pulse with 6 μJ energy and neglect loss for now. Supplementary Fig. 4 shows this comparison for the two cases, when ionization is switched off and when it is included.

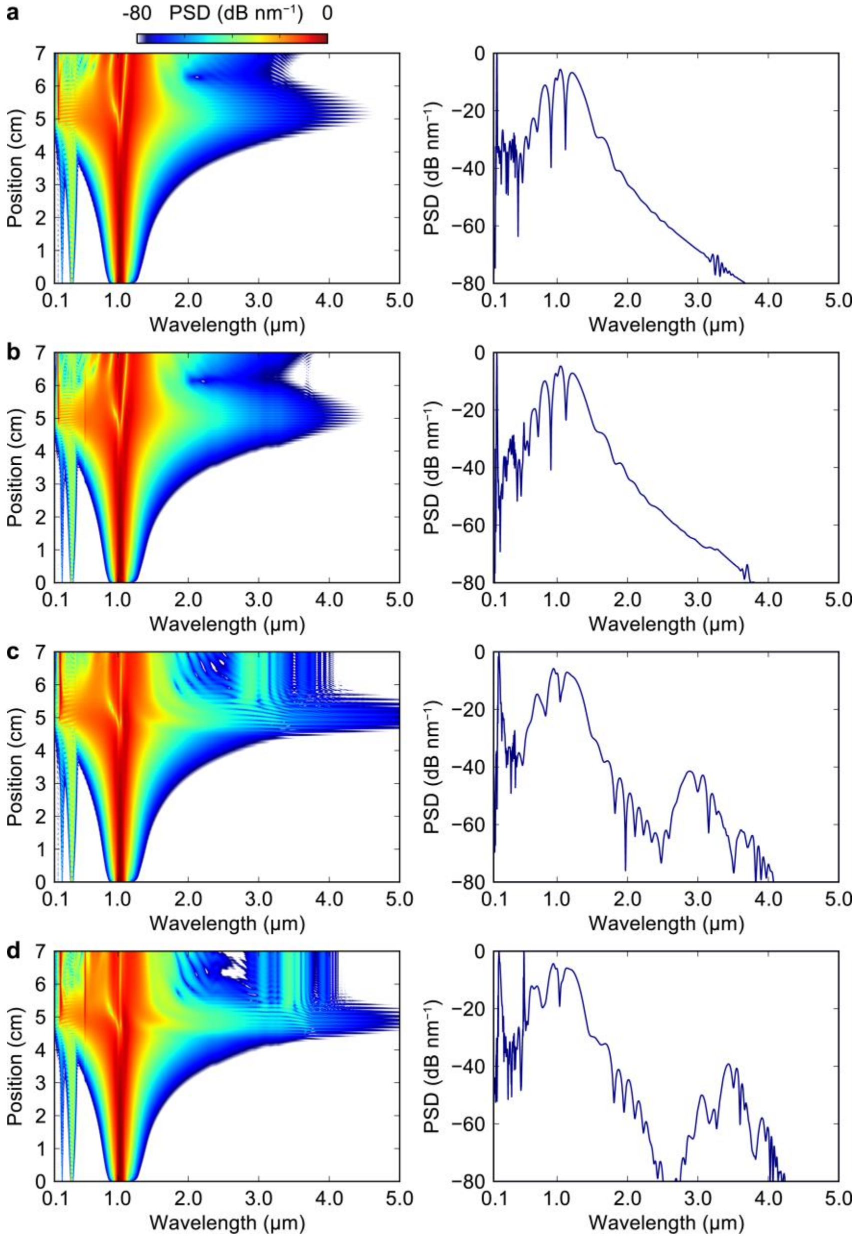


**Supplementary Figure 4 | Influence of the dispersion model on the mid-infrared dispersive wave emission.** Evolution of the power spectral density (PSD, normalized to the peak of the spectrum) with propagation distance. **a,** Capillary-like dispersion model, without ionization. **b,** Dispersion from finite element modelling, without ionization. **c,** Capillary-like dispersion model, with ionization. **d,** Dispersion from finite element modelling, with ionization. Loss was neglected in all simulations. The corresponding spectra on the right are at the fibre output.

When ionization is neglected, the simulation of the real fibre shows a weak narrowband enhancement of the spectrum at the core-wall resonance (Supplementary Fig. 4b), which is absent in the idealized case (Supplementary Fig. 4a). However, there is no trace of MIR DW emission. Only when ionization is included is a MIR DW generated, with a spectrum that is even more structured in the real fibre (Supplementary Fig. 4d) than in the ideal fibre (Supplementary Fig. 4c). As this structure comes from local changes in dispersion at the resonances, it persists even when the input energy is changed

(Supplementary Fig. 5), in agreement with the experimental observations made in the kagomé-PCF (Fig. 1) and the single-ring PCF. Since the imperfections in the real fibre structure cannot be perfectly modelled using FEM, the simulations can only provide qualitative agreement with the experiments. However, they capture well the spectral features observed in the experiments.

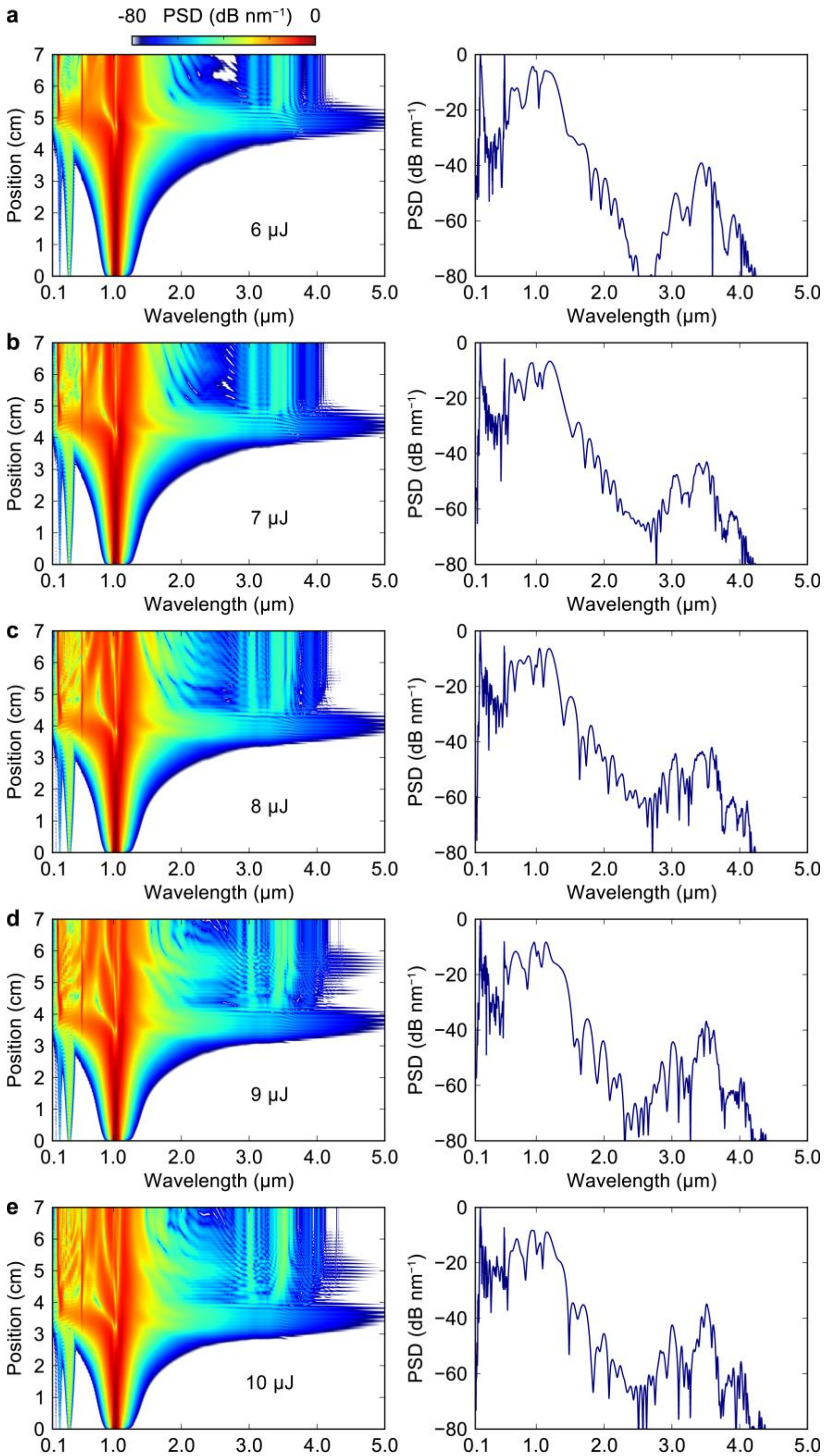


**Supplementary Figure 5 | Influence of the input energy on the mid-infrared dispersive wave emission.** Evolution of the power spectral density (PSD, normalized to the peak of the spectrum) with propagation distance for input energies from 6 to 10 μJ, including fibre dispersion from finite element modelling but neglecting loss. The corresponding spectra on the right are at the fibre output.

Supplementary Fig. 6 shows the influence of fibre loss on the MIR DW emission. When the loss is gradually increased to the value predicted by FEM, the signal of the MIR DW gets weaker and virtually disappears when the full loss is considered. Since, however, in the experiments, clear amplification of the MIR DW spectrum above the continuum tail is observed and the signal is approximately at the −40 dB level with respect to the pump when the DW is generated close to the output fibre end, we conclude that the FEM overestimates the fibre loss in the MIR. Already a loss that is 5 times lower than predicted by FEM gives emission of a MIR DW (note that, in this case, the loss is still above 1000 dB $m^{-1}$ for wavelengths beyond 3 μm). As the attenuation estimated by FEM mainly comes from confinement loss of the mode, the light is expected to leak out the fibre core (e.g. into the cladding), but is not strongly absorbed and can hence be collected using a high-numerical aperture mirror as in the experiments. Finally, from Supplementary Fig. 6 it is evident that the loss scales the amplitude of the MIR DW, but has only a minor influence on the spectral structure.

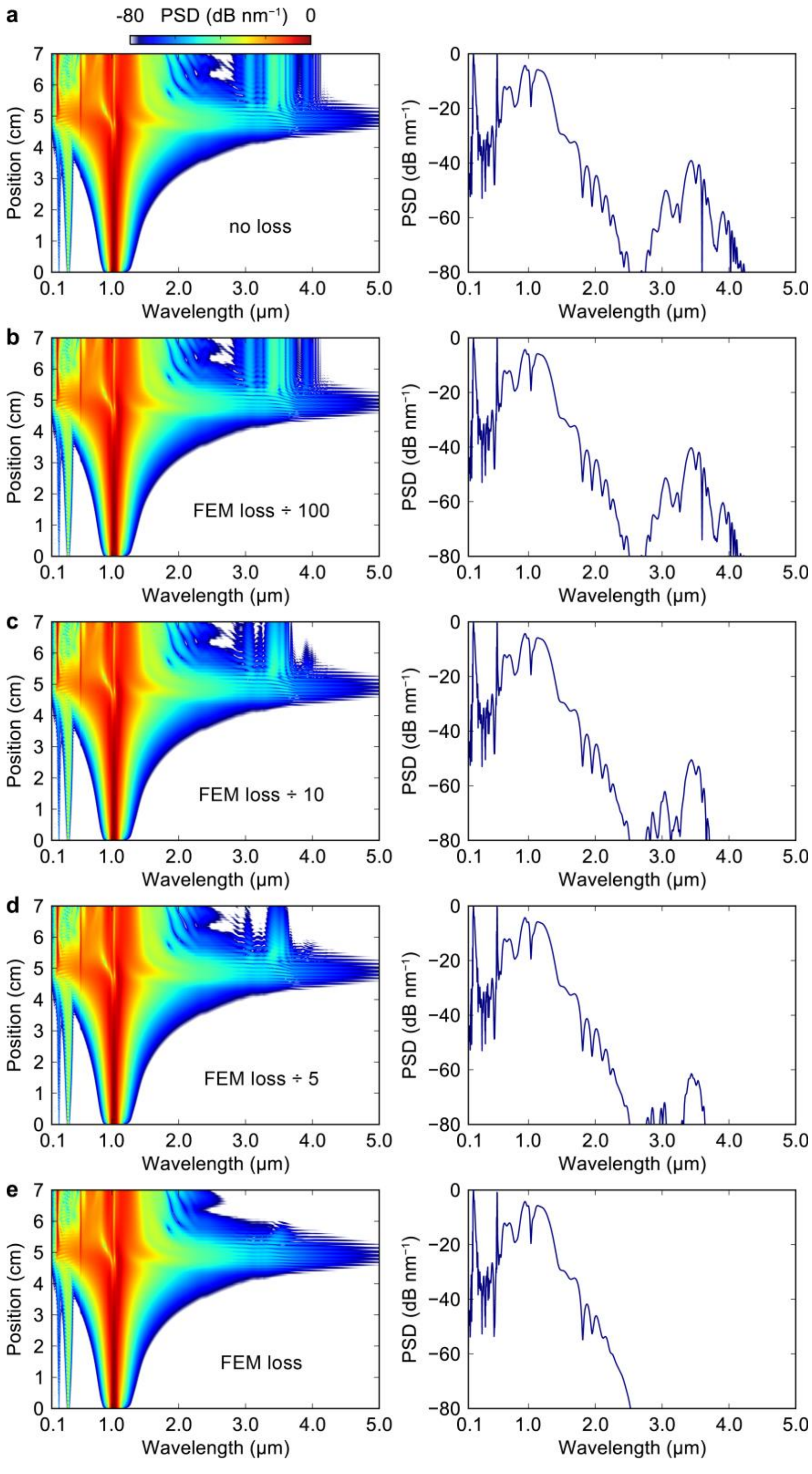


**Supplementary Figure 6 | Influence of fibre loss on the mid-infrared dispersive wave emission.** Evolution of the power spectral density (PSD, normalized to the peak of the spectrum), including fibre dispersion and loss from finite element modelling (FEM). In addition to the full loss, it was also scaled down by a factor of 5, 10, 100 and set to zero, respectively. The corresponding spectra on the right are at the fibre output.

## Supplementary Note 3

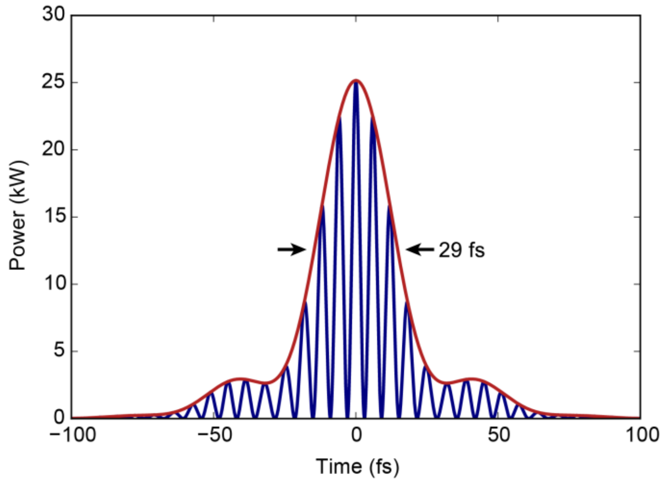


**Supplementary Figure 7 | Ultrashort mid-infrared pulses via dispersive wave emission.** Transform-limited pulse based on the mid-infrared dispersive wave between 3 and 4 μm. Fibre filled with 4 bar of argon, 6 μJ input energy. Spectrum (Fig. 1a) and energy (1 nJ) according to the experimental measurements. The transform-limited pulse duration is 29 fs (full-width-half-maximum).

## Supplementary Note 4

Unlike in the experiments, ionization can be switched off in numerical simulations (Supplementary Fig. 8). In this case, no mid-infrared dispersive wave is emitted since phase-matching cannot be fulfilled. Also, the peak power of the self-compressed pulse is higher as there are no ionization-related losses (which are apparent in the measured transmission of the fibre Fig. 1e). The presence of the plasma lowers the refractive index, leading to a blueshift and acceleration of the pulse in the anomalous dispersion region (Supplementary Fig. 8a, where the pulse curves to the left after the maximum temporal compression point). When ionization is switched off, however, the spectral recoil following self-compression and DW emission in the ultraviolet effectively red-shifts the pulse and it gets decelerated (Supplementary Fig. 8b).

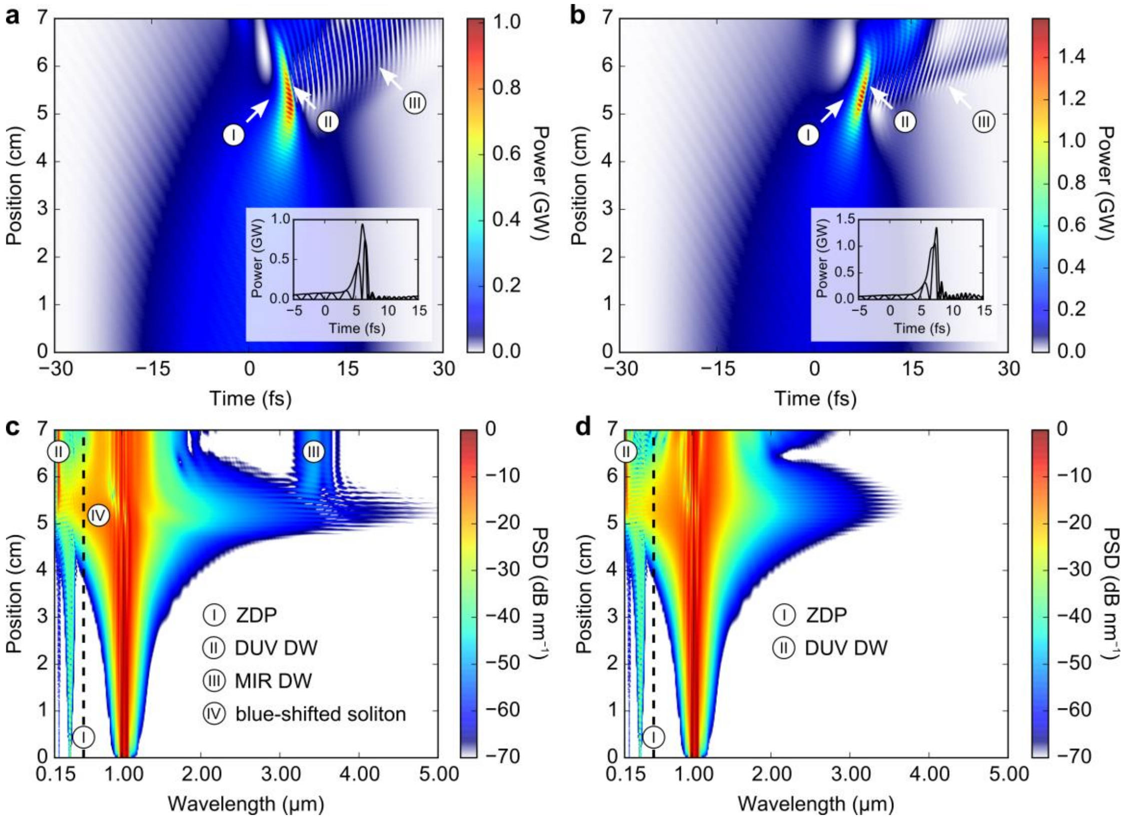


**Supplementary Figure 8 | Numerically simulated pulse propagation.** Fibre filled with 4 bar of argon, 6 μJ input pulses (measured via frequency-resolved optical gating, Supplementary Fig. 10). Evolution of the temporal pulse shape with propagation distance, when ionization is included (**a**) and when it is switched off (**b**). The inset shows the pulse at the maximum temporal compression point. (I)—maximum temporal compression point. (II)—shock front. (III)—deep ultraviolet dispersive wave (DUV DW). **c, d,** Evolution of the power spectral density (PSD, normalized to the peak of the spectrum) with propagation distance, when ionization is included (**c**) and when it is switched off (**d**). The dispersion is anomalous on the long-wavelength side of the zero dispersion point (ZDP). The dynamics are very similar in both cases, yet no mid-infrared (MIR) DW is emitted when ionization is switched off.

## Supplementary Methods

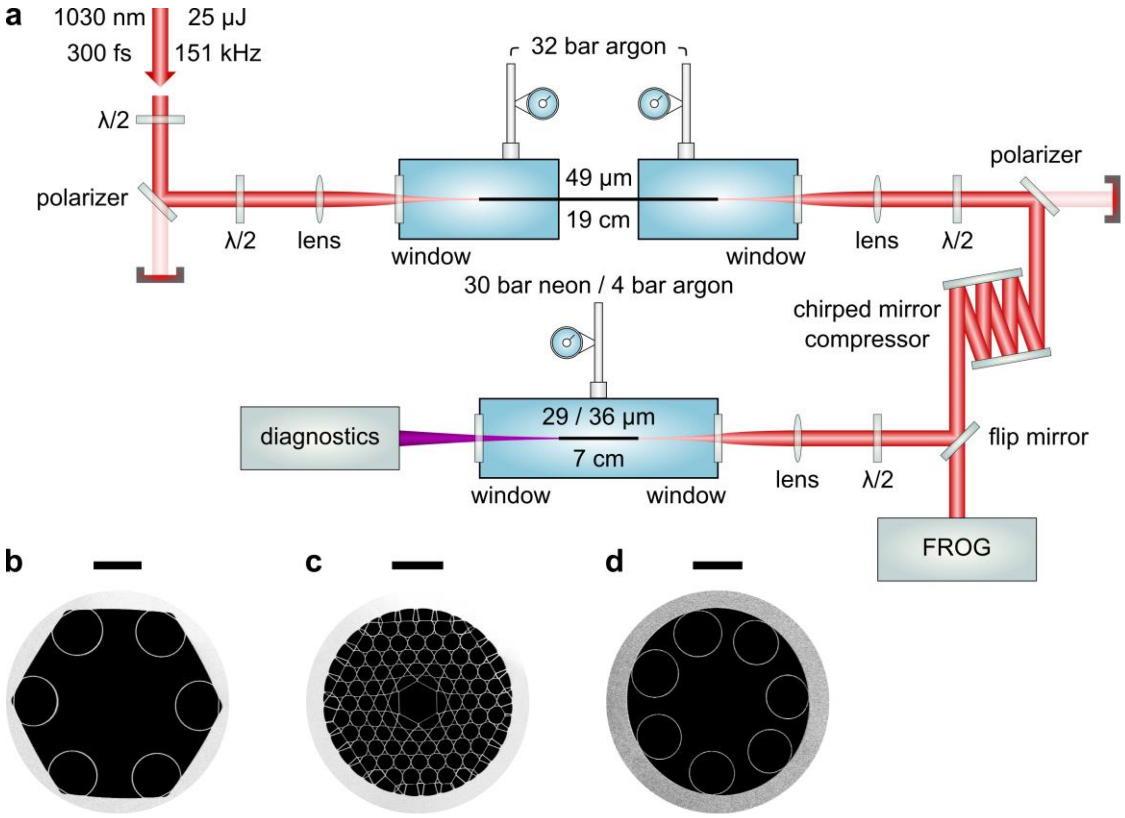


**Supplementary Figure 9 | Experimental setup. a,** The fibres are placed in gas cells filled without pressure gradients. Half-wave plates and polarizers are used for power control. Half-wave plates ($\lambda/2$) before the fibres are used to align the linear input polarization with the slightly birefringent axes of the fibres to optimize the polarization extinction ratio. FROG—frequency-resolved optical gating device. **b,** Scanning electron micrograph (SEM) of the single-ring photonic crystal fibre (PCF) with 49 µm core diameter, used in the first stage. The loss is ~1 dB $m^{-1}$ around the pump wavelength (1030 nm). The scale bar denotes 20 µm. **c,** SEM of the kagomé-PCF with 36 µm core diameter, used in the second stage. The loss is ~1 dB $m^{-1}$ around 1030 nm and below ~5 dB $m^{-1}$ over the wavelength range from 450 nm to 1.75 µm. The scale bar denotes 50 µm. **d,** SEM of the single-ring PCF with 29 µm core diameter, alternatively used in the second stage. The scale bar denotes 15 µm.

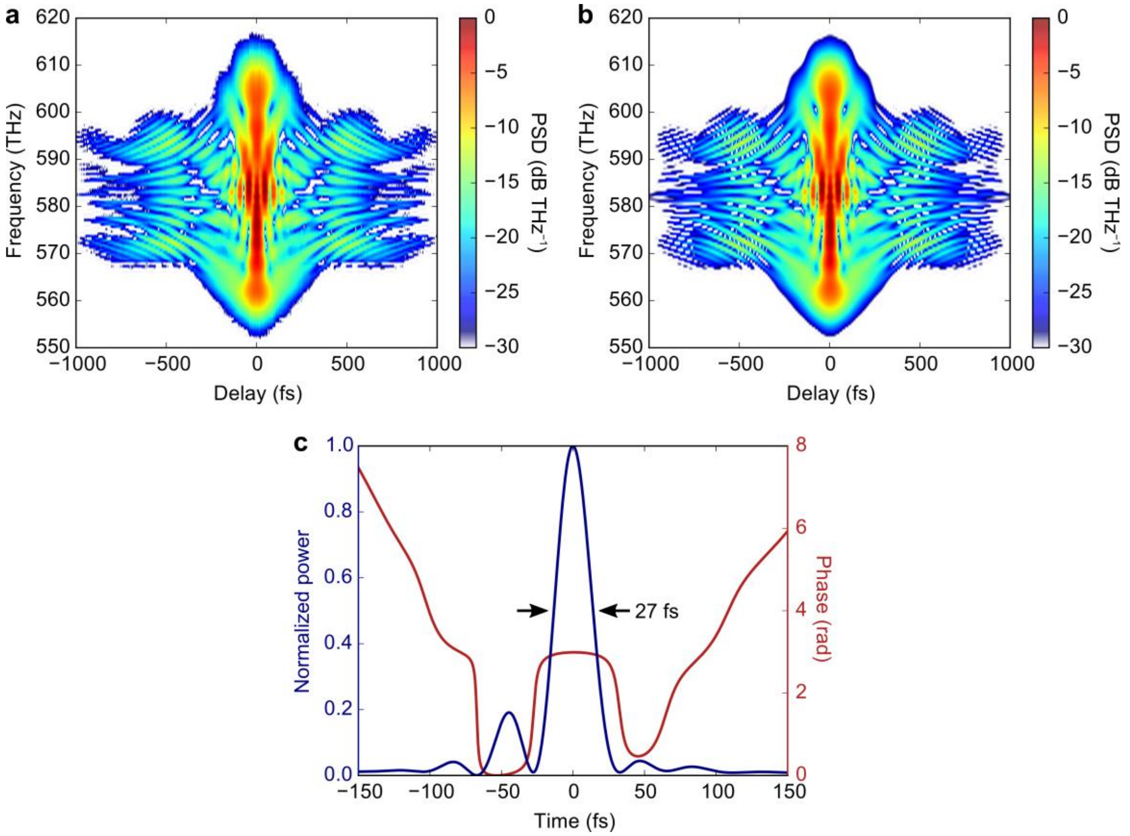


**Supplementary Figure 10 | Input pulse to the second fibre.** Second harmonic generation frequency-resolved optical gating measurement of the pulse after nonlinear compression. **a,** Measured trace. **b,** Retrieved trace. **c,** Pulse at the input of the second fibre (the dispersion of the half-wave plate, the lens and the gas cell window before the input of the second fibre was added numerically). Power (blue, normalized to its peak value) and phase (red). The full-width-half-maximum pulse duration is 27 fs.

The fibres used in the experiments guide via anti-resonant reflection, and, despite their different structure, can be treated identically in terms of guided modes and dispersion. The modal refractive index $n$ of the (gas-filled) fibres can be approximated to good accuracy by that of a capillary fibre[1]:

$$n_{mn}(\lambda, p, T) = \sqrt{n_{\text{gas}}^2(\lambda, p, T) - \frac{\lambda^2 u_{mn}^2}{4\pi^2 a^2(\lambda)}}, \tag{1}$$

where $\lambda$ is wavelength, $n_{\text{gas}}$ is the refractive index of the filling gas, $p$ is its pressure, $T$ its temperature, $a$ is the wavelength-dependent effective core radius of the fibre and $u_{mn}$ is the $n$th zero of the $m$th-order Bessel function of the first kind. Through careful launch alignment, we effectively excite only the fundamental $LP_{01}$-like mode, for which $m = 0$ and $n = 1$. A wavelength-dependent effective core radius is required to accurately describe the modal refractive index at longer wavelength. It is given by[2]:

$$a(\lambda) = \frac{a_0}{1 + s\lambda^2/(a_0 h)}, \tag{2}$$

where $a_0$ is the core radius of the fibre, $h$ is the core wall thickness and $s$ is a model parameter. For the kagomé-photonic crystal fibre used in the experiments, $a_0 = 18\,\mu\text{m}$, $h = 200\,\text{nm}$ and the model parameter $s = 0.08$ was determined from finite element modelling of an idealized fibre with the same

structural parameters. Supplementary Fig. 11 shows the group velocity dispersion of this fibre, calculated using Supplementary Eqs. (1) and (2).

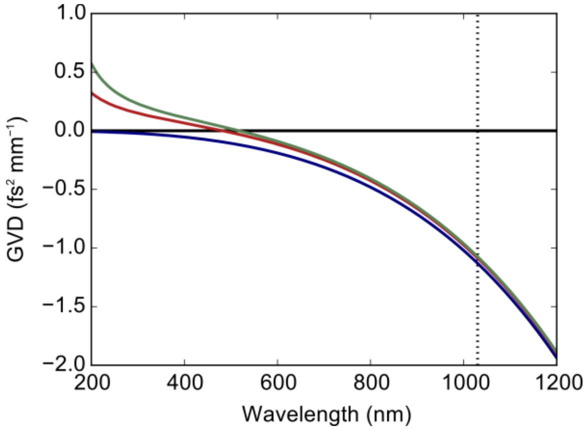


**Supplementary Figure 11 | Dispersion of the kagomé-photonic crystal fibre.** The group velocity dispersion (GVD) of the evacuated fibre (blue) is anomalous everywhere, and the normal dispersion of the filling gas (30 bar neon (red) or 4 bar argon (green)) shifts the zero dispersion point into the visible. The dotted line denotes the pump wavelength (1030 nm).

The pulse propagation inside the gas-filled fibre was numerically simulated using a single-mode unidirectional field equation[3]:

$$\frac{\partial \tilde{E}(z,\omega)}{\partial z} = \mathrm{i}\left(\beta(\omega) - \beta_1\omega\right)\tilde{E}(z,\omega) - \frac{\alpha}{2}\tilde{E}(z,\omega) + \mathrm{i}\frac{\omega^2}{2c^2\varepsilon_0\beta(\omega)}\tilde{P}_{\mathrm{NL}}(z,\omega), \tag{3}$$

where $\tilde{E}$ is the electric field in the frequency domain, $z$ is the position in the fibre (propagation distance), $\omega$ is frequency, $\beta$ is the propagation constant of the fibre mode (calculated using Supplementary Eqs. (1) and (2), or derived from finite element modelling), $\beta_1$ is the inverse of the group velocity at the frequency of the pump, $\alpha$ is the fibre loss (taken to be 1 dB m$^{-1}$, or derived from finite element modelling), $c$ is the speed of light in vacuum, $\varepsilon_0$ is the vacuum permittivity and $\tilde{P}_{\mathrm{NL}}$ is the nonlinear polarization in the frequency domain given by

$$\tilde{P}_{\mathrm{NL}}(z,\omega) = \mathrm{F}\left(\varepsilon_0\chi^{(3)}E(z,t)^3 + P_{\mathrm{ion}}(z,t)\right), \tag{4}$$

where $\mathrm{F}$ denotes the Fourier transform, $\chi^{(3)}$ is the third-order nonlinear susceptibility of the gas, $t$ is time with respect to a reference frame propagating with group velocity $1/\beta_1$ and $P_{\mathrm{ion}}$ is the plasma polarization. While the optical Kerr effect is governed by $\chi^{(3)}E(z,t)^3$, $P_{\mathrm{ion}}$ is given by[4]

$$\frac{\partial P_{\mathrm{ion}}(z,t)}{\partial t} = \frac{I_{\mathrm{P}}}{E(z,t)}\frac{\partial \rho(z,t)}{\partial t} + \frac{e^2}{m_{\mathrm{e}}}\int_{-\infty}^{t}\rho(z,t')E(z,t')\mathrm{d}t', \tag{5}$$

where $I_{\mathrm{P}}$ is the ionization potential of the gas, $\rho$ is the plasma density (calculated using the Perelomov, Popov, Terent'ev ionization rates[5], modified with the Ammosov, Delone, Krainov coefficients[6]) and $e$ and $m_{\mathrm{e}}$ are the charge and mass of the electron.

## Supplementary References


1. Travers, J. C., Chang, W., Nold, J., Joly, N. Y. & Russell, P. St.J. Ultrafast nonlinear optics in gas-filled hollow-core photonic crystal fibers [Invited]. *J. Opt. Soc. Am. B* **28,** A11–A26 (2011).
2. Finger, M. A., Joly, N. Y., Weiss, T. & Russell, P. St.J. Accuracy of the capillary approximation for gas-filled kagomé-style photonic crystal fibers. *Opt. Lett.* **39,** 821–824 (2014).
3. Tani, F., Travers, J. C. & Russell, P. St.J. Multimode ultrafast nonlinear optics in optical waveguides: numerical modeling and experiments in kagomé photonic-crystal fiber. *J. Opt. Soc. Am. B* **31,** 311–320 (2014).
4. Geissler, M. *et al.* Light Propagation in Field-Ionizing Media: Extreme Nonlinear Optics. *Phys. Rev. Lett.* **83,** 2930 (1999).
5. Perelomov, A. M., Popov, V. S. & Terent'ev, M. V. Ionization of Atoms in an Alternating Electric Field. *Sov. Phys. JETP* **23,** 924 (1966).
6. Ilkov, F. A., Decker, J. E. & Chin, S. L. Ionization of atoms in the tunnelling regime with experimental evidence using Hg atoms. *J. Phys. B: At. Mol. Opt. Phys.* **25,** 4005 (1992).
